# The Comb Jelly Opsins and the Origins of Animal Phototransduction

**DOI:** 10.1093/gbe/evu154

**Published:** 2014-07-24

**Authors:** Roberto Feuda, Omar Rota-Stabelli, Todd H. Oakley, Davide Pisani

**Affiliations:** ^1^Division of Biology and Biological Engineering, California Institute of Technology; ^2^Research and Innovation Centre, Fondazione Edmund Mach, San Michele all'Adige (TN), Italy; ^3^Department of Ecology, Evolution and Marine Biology, University of California, Santa Barbara; ^4^School of Biological Sciences and School of Earth Sciences, University of Bristol, United Kingdom

**Keywords:** opsin, comb jelly, evolution, vision, Metazoa, Ctenophora

## Abstract

Opsins mediate light detection in most animals, and understanding their evolution is key to clarify the origin of vision. Despite the public availability of a substantial collection of well-characterized opsins, early opsin evolution has yet to be fully understood, in large part because of the high level of divergence observed among opsins belonging to different subfamilies. As a result, different studies have investigated deep opsin evolution using alternative data sets and reached contradictory results. Here, we integrated the data and methods of three, key, recent studies to further clarify opsin evolution. We show that the opsin relationships are sensitive to outgroup choice; we generate new support for the existence of Rhabdomeric opsins in Cnidaria (e.g., corals and jellyfishes) and show that all comb jelly opsins belong to well-recognized opsin groups (the Go-coupled opsins or the Ciliary opsins), which are also known in Bilateria (e.g., humans, fruit flies, snails, and their allies) and Cnidaria. Our results are most parsimoniously interpreted assuming a traditional animal phylogeny where Ctenophora are not the sister group of all the other animals.

## Incongruences in Opsin and Animal Evolution

As G-protein-coupled receptors that mediate light detection across most animal lineages (Feuda et al. 2012; Rivera et al. 2012) opsins are key to understanding the origins and evolution of light sensitivity, eyes, and vision. Based on studies in bilaterian animals, opsins have been classified into three subfamilies: The ciliary (C–), rhabdomeric (R–), and Go-opsins (Terakita 2005). Opsins of these three subfamilies couple with different G-proteins allowing for the simultaneous existence of multiple light-dependent signaling pathways. Where known, C-opsins couple with G-proteins of the Gα (i/t)-type, Go-opsins usually couple with Gα (o) or Gα (s), and R-opsins with Gα (q) (Terakita 2005; Koyanagi et al. 2008). Many hypotheses of opsin evolution have been proposed, but consensus has remained elusive (e.g., Terakita 2005; Plachetzki et al. 2007; Suga et al. 2008; Porter et al. 2011; Feuda et al. 2012; Schnitzler et al. 2012). In particular, two recent studies analyzed complementary data sets, reaching very dissimilar conclusions with conflicting implications for opsin origins, and our understanding of early animal evolution

The first study by Feuda et al. (2012) found sequences from Placozoa (that they called “placopsins”) to be the sister of all known animal opsins, and consistent with other studies, they found melatonin receptors (MLTs, Fredriksson et al. 2003; Srivastava et al. 2010; Feuda et al. 2012) to be the closest outgroup to opsins + placopsins. Placopsins remain functionally uncharacterized, and because they lack the retinal-binding lysine, they might not function in light reception (Feuda et al. 2012). By using “Placopsins" and the MLTs (as outgroups to opsins), Feuda et al. found that known cnidarian opsins belong to one of the three known bilaterian opsin subfamilies (the C–, R–, or Go-opsins). R-opsins were previously unknown in Cnidaria, and no cnidarian opsin was yet known to couple with Gα (q), leaving some doubts about the nature of the sequences that Feuda et al. (2012) identified as R-opsins. However, a cnidarian opsin from the staghorn coral (*Acropora palmata*) has recently been shown to have an in vitro functional association with a putative Gα (q) (Mason et al. 2012). This suggests that this sequence (Acropsin3) might be a functional R-opsin, but its phylogenetic relationships remain uncertain. The scenario proposed by Feuda et al. (2012) to explain their results suggests that visual opsins evolved after Placozoa separated from Cnidaria and Bilateria but before the latter separated from each other. Feuda et al. (2012) did not have data for Ctenophora (i.e., the comb jellies). However, given previous phylogenomic results (Philippe et al. 2009, 2011; Dohrmann and Wörheide 2013; Nosenko et al. 2013) suggesting that Ctenophora, Cnidaria, and Bilateria are more closely related with each other than they are with the sponges and the Placozoa, they concluded that their results were compatible with a traditional view of animal evolution (an hypothesis we refer to as “Neuralia”). Differently from Nielsen (2012), Neuralia is here to be interpreted as simply stating that Bilateria, Cnidaria, and Ctenophora shared a common ancestor to the exclusion of the Placozoa and the sponges, irrespective of whether, within Neuralia, Cnidaria and Ctenophora form monophyletic Coelenterata (Philippe et al. 2009, 2011; Nosenko et al. 2013) or a paraphyletic group where Ctenophora is closer to Bilatera than it is to Cnidaria (Nielsen 2012).

The second recent study, by Schnitzler et al. (2012), analyzed a data set including three opsins from the genome of the ctenophore *Mnemiopsis leydi* (Ryan et al. 2013) and found one of these opsins (Mnemiopsis3) to emerge as the sister of all remaining animal opsins. These results can be considered to be consistent with analyses suggesting that Ctenophora are the sister group of all the other animals, rather than neuralians. A hypothesis we refer to as “Ctenophora-early” (Dunn et al. 2008; Hejnol et al. 2009; Ryan et al. 2013; Moroz et al. 2014). The results of Schnitzler et al. (2012), if correct, imply that opsins emerged in the stem animal lineage, that sponges have secondarily lost their opsins, and that the placopsins have secondarily lost their retinal-binding lysine.

## Understanding Opsin Evolution through Data and Methods Integration

We synthesized the studies of Feuda et al. (2012), Mason et al. (2012), and Schnitzler et al. (2012). These studies were published nearly contemporaneously and will benefit from the complementary nature of the data (see supplementary table S1, Supplementary Material online, for a list of all considered sequences and taxa) and analyses they presented. For example, a primary conclusion of Feuda et al. (2012)—that cnidarians possess all three subfamilies of known bilaterian opsins rests on the inclusion of two sequences from the cnidarian *Nematostella vectensis* (13116 and 33918) for which there is no clear evidence of expression and that seem to lack (at the least) a canonical start codon. Although functional cnidarian orthologs to *Nematostella* 13116 and 33918 were not available to Feuda et al. (2012), Acropsin3 (from the staghorn coral *A. palmata*) is now available. Importantly, its in vitro functional association with a putative Gα (q) is consistent with this gene being a functional R-opsin and including Acropsin3 in phylogenetic analyses will provide a key test of the hypothesis that cnidarians possess R-opsin orthologs. If *Acropsin3* will be found to cluster together with the putative R-opsins identified by Feuda et al. (2012), and if this group is found to represent the sister group of the bilaterian R-opsin, the confidence in the R-opsin nature of these cnidarian sequences will substantially increase. On the contrary, if Acropsin3 is not found to cluster with the putative cnidarian R-opsins identified by Feuda et al. (2012), our confidence on the existence of R-opsins in cnidarians will substantially decrease. Similarly, a primary conclusion of Schnitzler et al. (2012), that Mnemiopsis3 is the sister group of all animal opsins, rests on the assumption that their opsin topology is not affected by tree-reconstruction artifacts. Yet, it has been argued in a number of studies that ctenophorans rather than representing the sister group of all the other animals (Dunn et al. 2008; Hejnol et al. 2009; Ryan et al. 2013; Moroz et al. 2014) might simply be a fast-evolving neuralian lineage that emerges deeply in phylogenetic analyses when tree reconstruction artifacts are not corrected (Pick et al. 2010; Philippe et al. 2011; Dohrmann and Wörheide 2013; Nosenko et al. 2013). To minimize the impacts of tree reconstruction artifacts in data sets including fast-evolving sequences, the use of well-fitting substitution models and close outgroups are key (Rota-Stabelli and Telford 2008; Philippe et al. 2011; Feuda et al. 2012). However, Schnitzler et al. (2012) used a set of outgroups (the Muscarinic, acetylcholine, and somatostatin receptors) that are distantly related to the opsins. This was shown in previous analyses of the Rhodopsin-like GPCRs (Fredriksson et al. 2003; Srivastava et al. 2010; Feuda et al. 2012), which pinpointed the MLTs as the most likely outgroup of the opsin family. Further to that, Schnitzler et al. (2012) used a substitution model (WAG + G), which was shown by Feuda et al. (2012) not to fit opsin alignments well. Both these factors, which were addressed by Feuda et al. (2012), might have negatively influenced the analyses of Schnitzler et al. (2012). Interchanging the original outgrup sequences used by Schnitzler et al. (2012) with those of Feuda et al. (2012) and analyzing the resulting data set under GTR + G (as in Feuda et al. 2012) is key to test the claims of Schnitzler et al. (2012). Overall, the integrative approach taken in our study should allow a much better clarification of early opsin evolution.

## Outgroup Choice Is a Key Determinant of Ingroup Opsin Relationships

We began from two published data sets that we refer to as SEA Schnitzler et al. (2012) and FEA Feuda et al. (2012). We added new data to each and refer to the modified data sets by adding an “m” and a numerical index. We generated three data sets: SEAm1, SEAm2, and FEAm1. In SEAm1, we replaced the SEA’s original outgroups with the more closely related MLTs (Fredriksson et al. 2003; Srivastava et al. 2010; Feuda et al. 2012). In SEAm2, we added, as a second closely related outgroup, the Placopsins of Feuda et al. (2012). FEAm1 was generated adding to FEA all new ctenophoran (Schnitzler et al. 2012) and acroporan (Mason et al. 2012) opsins. Feuda et al. (2012) showed that GTR + G fits opsin alignments significantly better than any other available model including all empirical among-site heterogeneous models of the CAT-family (Lartillot and Philippe 2004; Quang et al. 2008). Here, we performed posterior predictive analyses of saturation to further test the fit of the GTR + G model to the data and evaluate whether this model adequately (sensu Goldman 1993) fits the data. This test showed that GTR + G quite faithfully predicts homoplasy in the opsin data, that is, it adequately fits the data and fits much better than the WAG + G model used by Schnitzler et al. (2012) (table 1 and supplementary fig. S1, Supplementary Material online).

**Table 1:** Posterior Predictive Analysis of Saturation

	Models					
	WAG			GTR		
	Observed	Predicted	P	Observed	Predicted	P
Substitutions	65.4727 ± 1.1915	63.8521 ± 1.5705	0.04	71.4523 ± 1.41065	71.5064 ± 1.73156	0.51
Homoplasy	52.4011 ± 1.13881	49.5447 ± 1.52248	0	58.443 ± 1.37283	57.4735 ± 1.7008	0.15

Note.—The difference in fit between the WAG and the GTR matrix to the opsin data is presented. It can be seen that under WAG, both the number of substitutions and the amount of homoplasy in the data are systematically underestimated and that the difference between observed and predicted homoplasy and substitutions are both significant. This indicate a poor fit of the of WAG + G model to the data. Differently, under GTR + G, both the observed substitutions and the homoplasy can be better predicted, and the difference between these values is never significant. See the PhyloBayes manual (Lartillot et al. 2007) for details about the posterior predictive test here performed, and see supplementary figure S1, Supplementary Material online, for a graphical representation of the results in this table.

**Fig. 1.—:**
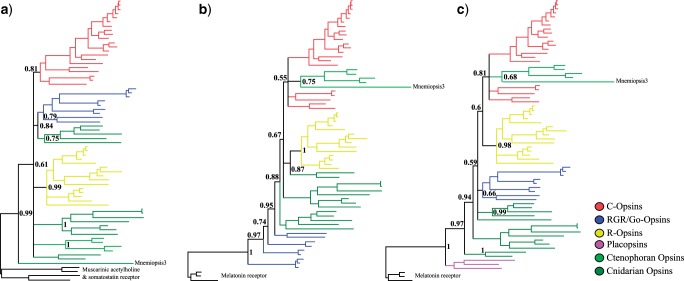
Results of the analyses of SEA, SEAm1, and SEAm2 under GTR + G. (*a*) Results of SEA original data set under GTR + G showing Mnemiopsis3 as the sister of all the other animal opsins. This is the same result that was obtained by Schnitzler et al. (2012) and indicates that model choice, GTR + G here and WAG + G in the study by Schnitzler et al. (2012), is not affecting tree reconstruction. (*b*) Results of the analysis of the SEA data set but using the MLTs as the only outgroups. In this tree, Mnemiopsis3 is not the sister group of all the other opsins, indicating the importance of outgroup selection in opsin analyses. (*c*) Results of the analysis of the SEA data set but using the MLTs and placozoans opsin-like sequences (Placopsins) as outgroups. Addition of the Placopsins does not change the relationships of Mnemiopsis3 but allow the recovery of a monophyletic Go-opsin group. Supplementary figure S2, Supplementary Material online, shows that the results of the data sets analyzed in figures 1*b* and *c* holds also under WAG + G.

**Fig. 2.—:**
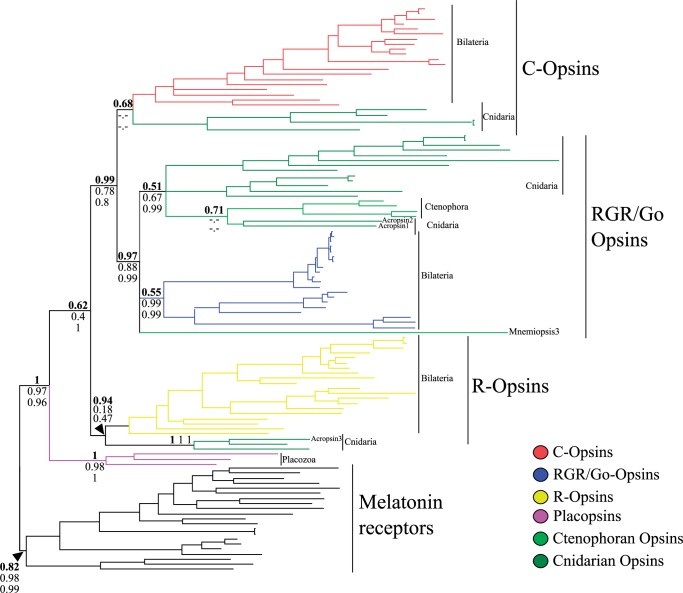
Results of the analyses of FEAm1 under GTR + G. The tree indicates that Mnemiopsis3 is not the sister group of all the other opsins, that Ctenophoran lost their R-opsins and most likely their C-opsins, and that Cnidarians possess R-opsins. Support values are from top to bottom PP (bold values), Sh-like bootstrap, and aBayes bootstrap. Supplementary figures S3, Supplementary Material online, show that the results obtained from the analysis of FEAm1 under GTR + G hold also under WAG + G.

We find that, despite differences in fit (see above), model choice did not affect the opsin phylogeny (compare fig. 1*b* and *c* with supplementary fig. S2*a* and *b*, Supplementary Material online, and fig. 2 with supplementary fig. S3, Supplementary Material online). Differently, outgroup choice had an important effect on the position of the critical Mnemiopsis3 gene. Figure 1*a* presents the tree obtained analyzing the original SEA data set under GTR + G. As pointed out above, even though GTR + G fits the data better than WAG + G (the model used by Schnitzler et al. 2012), the GTR + G and the WAG + G tree are the same. In contrast, our analyses of SEAm1 and SEAm2 show that outgroup choice dramatically affected phylogenetic inferences. When the MLTs are used as the outgroup (fig. 1*b*) the important Mnemiopsis3 gene does not emerge as the sister of all the other opsins. Instead, it emerges as the most divergent member (posterior probability [PP] = 0.75) of a ctenophoran-specific clade that includes all ctenophoran opsins. This ctenophoran-specific opsin group in then nested within the C-opsin subfamily (albeit with low support PP = 0.55). The further addition of the “Placopsins" (SEAm2, fig. 1*c*) results in the recovery of a monophyletic Go-opsin clade (PP = 0.66) and increases the support for an association of the ctenophoran-opsins with the C-opsins (*P* = 0.81). Figure 2 shows that also using FEAm1, Mnemiopsis3 does not emerge as the sister of all the other opsins. Instead, it appears as a divergent Go-opsin (PP = 0.97). For this data set, that we deem more reliable (see below the approximately unbiased [AU] test results), we also implemented sh-like bootstrap support values (SHB) and their Bayesian counterparts (aBayes support values [aBS]). Using SBH and aBS, support for Mnemiopsis3 as a Go-opsin is highly significant (0.88 and 0.99, respectively). All other ctenophoran opsins form a monophyletic group with as yet functionally uncharacterized cnidarian Go-opsins (PP = 0.51; SHB = 0.67; aBS = 0.99). Also for these sequences, the association with the Go-opsins is highly significant using the SHB and the aBS (fig. 2). The AU test (table 2), when applied to FEAm1, significantly rejected the possibility that Mnemiopsis3 could be the sister group of the other animal opsins (*P* = 0.005). This points out that FEAm1 is sufficiently informative to significantly differentiate alternative hypotheses of ctenophoran-opsins relationships. In contrast, when performed using SEA, SEAm1, and SEAm2, the AU test (table 2) proved indecisive, suggesting SAE does not convey a strong enough signal to allow the significant discrimination of alternative opsin phylogenies. Given that SEA does not seem to convey sufficient signal to discriminate between alternative hypotheses of ctenophoran-opsin relationships, we further focused on FEAm1 only and performed a posterior predictive analysis of composition, and a principal component analysis (PCA) of amino acid frequencies, to evaluate whether our results might have been affected by compositional biases. The posterior predictive analysis (supplementary table S2, Supplementary Material online) identified few compositionally heterogeneous sequences (*P* < 0.05). PCA (supplementary fig. S4, Supplementary Material online) shows that there is substantial homogeneity of composition among outgroups and other opsins, once the heterogeneous sequences in supplementary table S2, Supplementary Material online, are excluded. Outgroups sequences are well spread across the principal axis, albeit few outgroups form a tail. Irrespective of that, there is no clustering of outgroups and ingroup sequences, indicating that attraction artifacts (see Rota-Stabelli et al. 2013) should not affect our analyses that exclude sequences identified as heterogeneous by the posterior predictive analysis (reported in supplementary fig. S5, Supplementary Material online). Interestingly, this analysis (supplementary fig. S5, Supplementary Material online) identifies all ctenophore and cnidarian Go-opsins (including Mnemopsis3) as members of a monophyletic group. The same result is obtained (supplementary fig. S6, Supplementary Material online) when an analysis is performed that takes into account the covarion structure in the data (even though this analysis could not be run to convergence). This is what one would expect if Ctenophora were neuralians belonging to the traditionally recognized Coelenterata (i.e., Cnidaria plus Ctenophora—albeit the support for this group is not significant PP = 0.5). In addition, analyses of FEAm1 (fig. 2) further suggest that cnidarians have R-opsins, as the Gα (q)-binding Acropsin3 is found to cluster with the putative cnidarian R-opsins (PP = 0.94) of Feuda et al. (2012), and this result is invariant to the exclusion of compositionally heterogeneous opsin sequences (supplementary fig. S5, Supplementary Material online). However, lower SHB and aBS for this group (0.18 and 0.47, respectively, fig. 2) indicate that some instability affect this node. As more cnidarian opsins will become available in the future, the stability of this node could be further tested.

**Table 2:** AU Test Results

Hypothesis	Data Set			
	SEA	SEAm1	SEAm2	FEAm1
Mnemopsis3 is not the sister of all other opsins	0.437	0.228	0.297	0.995
Mnemopsis3 sister of all other opsins	0.563	0.772	0.703	0.005*

Note.—Topologies used for the AU test are those of figures 1 and 2 (in the case of SEA, SEAm1, SEAm2, and FEAm1, respectively). These trees were manually modified, by moving Mnemopsis3, to represent the alternative possible placement for this opsin sequence (in each considered case).

*Significant results.

## Opsins and Early Animal Evolution: Reciprocal Illumination

Our results show that the phylogenetic position of Mnemiopsis3 is outgroup dependent and sensitive to the inclusion of compositionally heterogeneous opsins in the data set. We conclude that the use of distant outgroups in Schnitzler et al. (2012) destabilized opsin ingroup relationships through the exacerbation of saturation-dependent artifacts, as shown previously for a different opsin data set (Plachetzki et al. 2007). Overall, our analyses suggest that cnidarians possess R-opsins (albeit this node is still somewhat unstable). This is because the acroporan opsin shown by Mason et al. (2012) to interact with Gα (q) groups with the putative R-opsins identified by Feuda et al. (2012). By turn, these opsins group with the Gα (q) binding, bilaterian R-opsins. We could not identify ctenophorans (or at the very least *M. leydi*) R-opsins. Because all other ctenophoran opsins emerge as either C or Go-opsins (depending on the data set used, figs. 1 and 2), a parsimonious interpretation is that the R-opsins and either the Go-opsins (according to SEAm, fig. 1) or most likely their C-opsins (as from the results of FEAm, fig. 2) have been lost or not yet detected in Ctenophora. These absences would represent secondary losses irrespective of whether Ctenophora-early or Neuralia is correct. However, more generally, our interpretation of the evolutionary history of opsin gene duplications and deletions depends on a correct interpretation of the relationships of the nonbilaterian animals. If Ctenophora-early is correct, R–, C–, and Go-opsins emerged in the stem-metazoan lineage. After that, a secondary (lineage specific) deletion would have caused ctenophorans to lose their R-opsins and either their C– or Go-opsins (figs. 1 and 2). In addition, under the Ctenophora-early hypothesis, sponges must have secondarily lost all their opsins, whereas Placozoa retained a divergent type of opsin (that might not function in light detection—the Placopsins). This scenario is not particularly parsimonious. Differently, if Neuralia is correct, as proposed in Feuda et al. (2012) scenario, C–, R–, and Go-opsins emerged in the stem neuralian ancestor, sponges never had opsins, and the placopsins represent the sister group of all other animal opsins (a more parsimonious reconstruction). The discovery of a Ctenophora-specific opsin found to be the sister of all the other opsins, as in Schnitzler et al. (2012), might be seen as evidence corroborating the scenario underpinned by the Ctenophora-early hypothesis. However, this could only be the case if Ctenophora were also shown not to have opsins belonging to the bilaterian subfamilies (C–, Go-, and R–), which is not the case when using close opsin outgroup genes, as ctenophorans have opsins belonging to the C + Go Cluster (Schnitzler et al. 2012) and figure 1*a*. It follows that the “basal” position of Mnemopsis3 in Schnitzler et al. (2012) and in figure 1*a* is better seen as a possible tree-reconstruction artifact. Indeed, if ctenophores are fast evolving (Pick et al. 2010; Philippe et al. 2011; Dohrmann and Wörheide 2013; Nosenko et al. 2013), and precautions are not taken to avoid tree reconstruction artifacts, their most divergent opsins (e.g., Mnemiopsis3) would be expected to cluster at the base of the opsin tree.

To minimize attraction artifacts, outgroup choice is key. Schnitzler et al. (2012) used outgroups that are not closely related to the opsin family (Fredriksson et al. 2003; Srivastava et al. 2010; Feuda et al. 2012). Our results, derived using close opsin outgroups (MLTs and placopsins, Fredriksson et al. 2003; Srivastava et al. 2010; Feuda et al. 2012), corroborate the view that Mnemiopsis3 is a divergent (i.e., fast evolving) opsin of bilaterian type (either a Go- or a C-opsin), not the sister of all other animal opsins. Our results show that opsins underwent a series of duplications before the separation of Cnidaria, Ctenophora, and Bilateria (as postulated by Feuda et al. (2012)). After that, Ctenophora (or at the least *M. leydi*) lost their R-opsins and either their C– (figs. 2 and supplementary figs. S3 and S5, Supplementary Material online) or less likely their Go-opsins (fig. 1).

Results of the analyses of a single protein family cannot represent a test of the animal phylogeny. Therefore, whether the animal opsins emerged in a stem metazoan (as implied by Schnitzler et al. 2012) or in a stem neuralian (as suggested by Feuda et al. 2012) remains unclear. Nevertheless, given the lack of opsins in sponges, lack of a retinal-binding lysine in the placopsins, and the clustering of cnidarian and ctenophoran sequences in figure 2 and supplementary figures S3 and S5, Supplementary Material online, it is clear that opsin evolution fits best a traditional scenario of animal relationships where Ctenophora are neuralians and not the sister group of all other animals.

## Materials and Methods

### Data Sets Generation

The data sets of Feuda et al. (2012) and of Schnitzler et al. (2012) were modified (updated) as necessary, generating the FEAm1 and SEAm1 and SEAm2 alignments—all available as supplementary material, Supplementary Material online. In the case of Feuda et al. (2012) data set, all the ctenophoran opsins identified by Schnitzler et al. (2012) and the cnidarian opsins identified by Mason et al. (2012) in the acroporan *A. palmata* were added to the alignment (generating FEAm1). Inclusion of acroporan sequences is key to test the R-opsin nature of the putative R-opsins of Feuda et al. (2012), see above. Ctenophoran opsins have also been added to Feuda et al. (2012) data set to further test the nature of these sequences, and the stability of the results obtained from the analyses of FEA as new data are included. In the case of Schnitzler et al. (2012) data set, we created two updated data sets (SEAm1 and SEAm2). In both SEAm1 and SEAm2, the original outgroups were deleted. In SEAm1, the MLTs, identified by Feuda et al. (2012), Fredriksson et al. (2003), and Srivastava et al. (2010) to represent one of the closest outgroups of the opsin family (if not the closest one), was used. In SEAm2, both the MLTs and the opsin-like sequences identified by Feuda et al. (2012) in Placozoa (i.e., the placopsins) were used as outgroups. In all cases, new sequences were added to the original data sets using the profile alignment option in MUSCLE (Edgar 2004). This was done to maintain comparability between the original results of Feuda et al. (2012) and Schnitzler et al. (2012) and those in this study. The final alignments were further manually adjusted (if necessary, e.g., to remove sites at the 3′- and 5′-end of the alignment present only in the newly added sequences).

### Phylogenetic Analyses

All three considered data sets (see above) were subjected to Bayesian analyses in PhyloBayes (Lartillot et al. 2009). All analyses were performed under the GTR + G and the WAG + G models. In addition, an analysis of the original SEA alignment was performed using the GTR + G model. For all analyses, two runs were performed, and convergence was tested using the BPCOMP program, which is part of PhyloBayes. All analyses were run to convergence (number of generations changed from analyses to analyses), and majority rule consensus trees were derived from the trees saved after convergence. Analyses were assumed to have converged when the standard deviation of the split frequencies between the trees in the compared runs dropped below 0.2 (see PhyloBayes manual).

FEAm1 was subjected to posterior predictive analyses of saturation (in PhyloBayes) under both GTR + G and WAG + G. Posterior predictive analyses allow evaluating how well a model fits a data set, rather then simply testing which model fits the data best. The second question (which model fits the data better between GTR + G and WAG + G) has already been addressed by Feuda et al. (2012), who showed that GTR + G provides a better fit to the data than other site homogeneous models like WAG + G and site-heterogeneous models of the CAT family (Lartillot and Philippe 2004; Quang et al. 2008). However, whether GTR + G (and WAG + G for that matter) fits the data adequately has never been investigated. Testing adequacy of fit (in addition to testing what is the best fitting model) is important as the best fitting model could still not fit the data adequately (Goldman 1993), and the use of models that do not fit the data adequately can drive the appearance of tree reconstruction artifacts.

The AU test was used (on SEA, SEAm1, SEAm2, and FEAm1) to evaluate whether these data sets could significantly discriminate between alternative hypotheses of ctenophoran opsin relationships. To calculate the AU test, we first used RAxML (Stamatakis 2006) to estimate site-wise likelihoods (for all positions in the considered alignments) under each considered alternative hypothesis, using the GTR + G model. The site-wise likelihood values were inputted to CONSEL (Shimodaira and Hasegawa 2001) to calculate the AU test. For the FEAm data set, the three in figure 2 was compared with one in which Mnemopsis3 was moved to represent the sister group of all the other opsins. For the SAE data sets, the topology of figure 1*A* was contrasted against the one in figure 1*B* and *C* (where all Ctenophoran opsins form a single group). To further test robustness of our results, for the FAEm1 data set, we also calculated node-specific SH-support values and their Bayesian counterparts (aBayes) support values (Anisimova et al. 2011) as implemented in PhyML (Guindon et al. 2010). Because of software limitations, these tests could only be performed using the WAG + G model. However, this should not be a problem as we showed that model choice was not a major determinant of the opsin relationships (see Results).

To test whether the results of our analyses could have been driven by compositional biases in the data, a posterior predictive analysis of composition was performed in PhyloBayes (under GTR + G) for FEAm1. Results of this test were used to identify and exclude from the alignment compositionally heterogeneous sequences. Analyses were repeated, for this reduced data set, under GTR + G in PhyloBayes and the results of this final analysis were compared against those obtained for the complete data set. Further to that, a PCA of the frequencies of the 20 amino acids in the remaining (compositional homogeneous) sequences of supplementary figure S4, Supplementary Material online, was performed. The first two axes, which overall describe 42% of compositional diversity, were plotted.

## Supplementary Material

Supplementary tables S1 and S2 and figures S1–S6 are available at *Genome Biology and Evolution* online (http://www.gbe.oxfordjournals.org/).

Supplementary Data
