# Supplementary material for: The Comb Jelly Opsins and the Origins of Animal Phototransduction
Source: Genome Biol Evol. 2014 Jul 24;6(8):1964–71. doi: 10.1093/gbe/evu154 (PMC4159004; doi:10.1093/gbe/evu154)
Supplement: Supplementary Data [file supp_6_8_1964__index.html]

The comb jelly opsins and the origins of animal phototransduction — The Comb Jelly Opsins and the Origins of Animal Phototransduction — Supplementary Data 

# The Comb Jelly Opsins and the Origins of Animal Phototransduction

## Supplementary Data

files

**Files in this Data Supplement:**

- Supplementary Data - zip file
