# Supplementary material for: The Comb Jelly Opsins and the Origins of Animal Phototransduction
Source: Genome Biol Evol. 2014 Jul 24;6(8):1964–71. doi: 10.1093/gbe/evu154 (PMC4159004; doi:10.1093/gbe/evu154)
Supplement: Supplementary Data [file supp_evu154_suppl_data.zip › Supplementary_On_Line.pdf]

The comb jelly opsins and the origins of phototransduction

Feuda Roberto, Omar Rota-Stabelli, Oakley Todd and Pisani Davide

Supplementary on line material

Table S1

A comparison of the sequences in FEAm and SEA

Legenda: X = The sequence is present in the data set; - = The sequence is not present in the data set. + = The sequence is not present in the data set but the effect of its inclusion has been previously tested (in Feuda et al. 2013).

Sequence ID	Taxon	FEM	SEA
Mnemiopsis_opsin3_215412	Ctenophore	X	X
Nematostella_94740	Cnidaria (Anthozoa)	X	X
Nematostella_208454	Cnidaria (Anthozoa)	+	X
Pleurobrachia_opsin1_CU419614	Ctenophore	X	X
Mnemiopsis_opsin1_13055	Ctenophore	X	X
Pleurobrachia_opsin2_FQ011385	Ctenophore	X	X
Mnemiopsis_opsin2_12047	Ctenophore	X	X
Nematostella_131013	Cnidaria (Anthozoa)	+	X
Nematostella_96290	Cnidaria (Anthozoa)	+	X
Nematostella_85309	Cnidaria (Anthozoa)	+	X
Nematostella_130042	Cnidaria (Anthozoa)	+	X
Geotria_RhB_AAR14683	Vertebrate	+	X
Geotria_RhA_AAR14682	Vertebrate	+	X
Platynereis_c-opsin_AAV63834	Anellida	+	X
Schistosoma_opsin_AAF73286	Platyhelminthes	+	X
Anopheles_r-opsin1_XP001238567	Arthropod	X	X
Platynereis_r-opsin_CAC86665	Anellida	X	X
Octopus_r-opsin_P09241	Mollusca	+	X
Euprymna_opsin_ACB05672	Mollusca	+	X
Loligo_f_opsin_P24603	Mollusca	+	X
Tra_429092_no_comment	Placozoa	X	-
Tra_429091_no_comment	Placozoa	X	-
Tra_430364_no_comment	Placozoa	X	-
NV_CN151Suga08_/1-24	Cnidaria (Anthozoa)	X	-
NV_CN146Suga08_/1-24	Cnidaria (Anthozoa)	X	-
OG28OrylaM/2-244	Arthropod	X	-
OG41BraflM/1-245	Chordata	X	-
OG7PlatynP/3-249	Annelida	X	-
OG18SchimP/1-241	Platyhelminthes	X	-

205NeooeL1/3-223	Arthropoda	X	-
054NeooeL1/53-287	Arthropoda	X	-
204NeooeL1/3-237	Arthropoda	X	-
004HelerRE/6-241	Arthropoda	X	-
010PapglRE/18-252	Arthropoda	X	-
033DianiRE/4-239	Arthropoda	X	-
045AnogaRE/2-237	Arthropoda	X	-
077DromeOC/1-236	Arthropoda	X	-
165BrakuM1/53-248	Arthropoda	X	-
090DappuM2/1-239	Arthropoda	X	-
150DappuU1/1-239	Arthropoda	X	-
109BomimUV/48-285	Arthropoda	X	-
120PieraUV/1-239	Arthropoda	X	-
149TrigrU1/1-239	Arthropoda	X	-
128ApomoBL/1-238	Arthropoda	X	-
162DappuR7/1-240	Arthropoda	X	-
Tra_429091jgi|T/1-23	Placozoa	X	-
Tra_429092jgi|T/1-19	Placozoa	X	-
Tra_430364jgi|T/1-22	Placozoa	X	-
010RGRDare/1-234	Vertebrate	X	-
012RGRGaga/1-234	Vertebrate	X	-
011hypXetr/1-234	Vertebrate	X	-
018PerMumu/11-260	Vertebrate	X	-
014PerBota/10-259	Vertebrate	X	-
015PerCafa/10-259	Vertebrate	X	-
019PerDare/10-259	Vertebrate	X	-
013OpsBrbe/5-258	Chordata	X	-
021NeuDare/1-252	Vertebrate	X	-
022NeuTagu/4-255	Vertebrate	X	-
023NeuGaga/4-255	Vertebrate	X	-
024NeuMumu/1-198	Vertebrate	X	-
025NeuRano/7-244	Vertebrate	X	-
029NeuCafa/1-252	Vertebrate	X	-
030NeuEqca/1-252	Vertebate	X	-
027NeuPatr/1-252	Vertebrate	X	-
028NeuHosa/1-252	Vertebrate	X	-
026NeuBota/1-250	Vertebrate	X	-
031NeuOran/1-252	Vertebrate	X	-
032NeuModo/1-252	Vertebrate	X	-
173RHOMumu/1-247	Vertebrate	X	-
170RHOOrcu/1-247	Vertebrate	X	-
156RHOLeja/1-247	Vertebate	X	-
158RHOSasa/1-247	Vertebrate	X	-

148RHOGaga/1-247	Vertebrate	X	-
135SWSOrla/1-247	Vertebate	X	-
208SWSTagu/46-291	Vertebate	X	-
204SWSNefo/1-247	Vertebate	X	-
217BluTaru/44-289	Vertebate	X	-
211SWSDare/45-290	Vertebate	X	-
128SWSPore/31-276	Vertebate	X	-
126SWSOrla/31-275	Vertebate	X	-
059LWSGeau/18-264	Vertebrate	X	-
043uppTeni/1-245	Vertebate	X	-
054VAoDare/1-250	Vertebrate	X	-
041ParXetr/1-250	Vertebrate	X	-
233TMTDare/1-227	Vertebrate	X	-
228EncDare/1-247	Vertebrate	X	-
Plos1_1/19-255	Cnidaria (Anthozoa)	X	-
Plos1_3/1-234	Cnidaria (Anthozoa)	X	-
Plos1_2/1-247	Cnidaria (Anthozoa)	X	-
NV_CN158Suga08_/1-24	Cnidaria (Anthozoa)	X	-
NV_CN152Suga08_/1-24	Cnidaria (Anthozoa)	X	-
OG50DappuT/1-253	Arthropod	X	-
OG45ApimeT/17-269	Arthropod	X	-
NV_CN144Suga08_/1-23	Cnidaria (Anthozoa)	X	-
NV_CN145Suga08_/1-23	Cnidaria (Anthozoa)	X	-
NV_CN137Suga08_/1-24	Cnidaria (Anthozoa)	X	-
NV_CN143Suga08_/1-24	Cnidaria (Anthozoa)	X	-
NV_CN135_Suga08/1-24	Cnidaria (Anthozoa)	X	-
ClR_CN108Suga08/1-19	Cnidaria (Hydrozoa)	X	-
CL_CN168_A9cr40/1-23	Cnidaria (Hydrozoa)	X	-
ClR_CN116Suga08/1-23	Cnidaria (Hydrozoa)	X	-
ClR_CN120Suga08/1-23	Cnidaria (Hydrozoa)	X	-
HM_CN131_221128/1-20	Cnidaria (Hydrozoa)	X	-
HM_CN170_UPI000/1-23	Cnidaria (Hydrozoa)	X	-
ClR_CN101Suga08/1-24	Cnidaria (Hydrozoa)	X	-
CR_CN100_Koyana/1-24	Cnidaria (Cubozoa)	X	-
acropsin2/1-248_no_c	Cnidaria (Anthozoa)	X	-
acropsin1/1-240_no_c	Cnidaria (Anthozoa)	X	-
acropsin3/1-252_no_c	Cnidaria (Anthozoa)	X	-
MTR1C_XENL/7-188	Melatonin receptor (outgroup)	X	-
UPI0001560/4-204	Melatonin receptor (outgroup)	X	-
MLT_B2Y4M8_/1-198	Melatonin receptor	X	-

	(outgroup)		
MLT_O88495_/1-202	Melatonin receptor (outgroup)	X	-
Tra_434217jgi|T/1-22	outgroup- Placozoa	X	-
Tra_435667jgi|T/1-22	outgroup- Placozoa	X	-
Tra_435668jgi|T/1-21	outgroup- Placozoa	X	-
Tra_435650jgi|T/1-22	outgroup- Placozoa	X	-
Tra_435652jgi|T/1-21	outgroup- Placozoa	X	-
Tra_435654jgi|T/1-21	outgroup- Placozoa	X	-
Tra_435653jgi|T/1-21	outgroup- Placozoa	X	-
Tra_435656jgi|T/1-21	outgroup- Placozoa	X	-
Tra_435655jgi|T/1-22	outgroup- Placozoa	X	-
Tra_435633jgi|T/1-20	outgroup- Placozoa	X	-
Tra_435634jgi|T/1-16	outgroup- Placozoa	X	-
Tra_435635jgi|T/1-22	outgroup- Placozoa	X	-
NEM_426957_jgi|/1-22	outgroup- Cnidaria	X	-
NEM_445570_jgi|/1-22	outgroup- Cnidaria	X	-
NEM_444070_jgi|/1-22	outgroup- Cnidaria	X	-
Nematostella_202741	Cnidaria (Anthozoa)	-	X
Mus_RGR_NP067315	Vertebrate	-	X
Homo_RGR_P47804	Vertebrate	-	X
Bos_RGR_NP786969	Vertebrate	-	X
Nematostella_199627	Cnidaria (Anthozoa)	-	X
Nematostella_123451	Cnidaria (Anthozoa)	-	X
Nematostella_214772	Cnidaria	-	X
Nematostella_214775	Cnidaria	-	X
Nematostella_95791	Cnidaria	-	X
Branchiostoma_Go-opsin_ABO50606	Chordata	-	X
Patinopecten_Go-opsin_AB006455	Mollusca	-	X
Nematostella_33918	Cnidaria (Anthozoa)	-	X
Nematostella_13116	Cnidaria (Anthozoa)	-	X
Mus_peropsin_NP033128	Vertebrate	-	X
Homo_peropsin_NP006574	Vertebrate	-	X
Branchiostoma_opsin6_AB050611	Chordata	-	X
Anopheles_pteropsin_XM312503	Arthropod	-	X
Apis_pteropsin_NP001035057	Arthropod	-	X
Xenopus_parapinopsin_AB159672	Vertebrate	-	X
Homo_MWS_NP000504	Vertebrate	-	X
Gallus_pinopsin_U15762	Vertebrate	-	X
Geotria_SWS1_AAR14684	Vertebrate	-	X
Homo_blue_opsin_M13299	Vertebrate	-	X
Rana_SWS1_BAA96828	Vertebrate	-	X
Xenopus_opsin_P29403	Vertebrate	-	X

Bos_rhodopsin_62460472	Vertebrate	-	X
Mus_rhodopsin_NP663358	Vertebrate	-	X
Homo_rhodopsin_NP000530	Vertebrate	-	X
Gallus_rhodopsin_NP001025777	Vertebrate	-	X
Geotria_SWS2_AAR14681	Vertebrate	-	X
Xenopus_green-rod_opsin_AAO38746	Vertebrate	-	X
Gallus_blue_sensitive_opsin_NP990848	Vertebrate	-	X
Homo_encephalopsin_NP055137	Vertebrate	-	X
Mus_encephalopsin_NP034228	Vertebrate	-	X
Homo_neuropsin_AY377391	Vertebrate	-	X
Danio_neuropsin_BX088599	Vertebrate	-	X
Anopheles_r-opsin2_XP556823	Arthropod	-	X
Drosophila_opsin_K02315	Arthropod	-	X
Drosophila_r-opsin6_Z86118	Arthropod	-	X
Xenopus_melanopsin_AAC41235	Vertebrate	-	X
Gallus_melanopsin_NP001038118	Vertebrate	-	X
Homo_melanopsin_AF147788	Vertebrate	-	X
Mus_melanopsin_NP038915	Vertebrate	-	X
Loligo_s_opsin_Q17094	Mollusca	-	X
Homo_5SR_BAA04107	somatostatin receptor (outgroup)	+	X
Drosophila_AR_AAF05299	allatostatin receptor (outgroup)	+	X
Homo_MAR_CAA68560	muscarinic acetylcholine receptor (outgroup)	+	X
Homo_5SR_BAA04107	somatostatin receptor (outgroup)	+	X

Table S2. Posterior predictive analysis of composition

taxon	p-value	z-score
NV_CN151Suga08_/1-24	0.809	-0.854
NV_CN146Suga08_/1-24	0.375	0.113
OG28OrylaM/2-244	0.395	0.065
OG41BraflM/1-245	0.918	-1.185
OG7PlatynP/3-249	0.169	0.878
OG18SchimP/1-241	0.916	-1.206
205NeooeL1/3-223	0.984	-1.612
054NeooeL1/53-287	0.357	0.21
204NeooeL1/3-237	0.282	0.502
004HelerRE/6-241	0.642	-0.492
010PapglRE/18-252	0.644	-0.492
033DianiRE/4-239	0.742	-0.672
045AnogaRE/2-237	0.583	-0.378
077DromeOC/1-236	0.766	-0.783
165BrakuM1/53-248	0.875	-1.051
090DappuM2/1-239	0.531	-0.221
150DappuU1/1-239	0.619	-0.453
109BomimUV/48-285	0.988	-1.549
120PieraUV/1-239	0.624	-0.491
149TrigrU1/1-239	0.642	-0.482
128ApomoBL/1-238	0.959	-1.372
162DappuR7/1-240	0.837	-0.913
Tra_429091jgi|T/1-23	0.158	1.07
Tra_429092jgi|T/1-19	0.529	-0.248
Tra_430364jgi|T/1-22	0.393	0.147
010RGRDare/1-234	0.407	0.124
012RGRGaga/1-234	0.332	0.302
011hypXetr/1-234	0.099	1.334
018PerMumu/11-260	0.441	-0.089
014PerBota/10-259	0.097	1.318
015PerCafa/10-259	0.135	1.032
019PerDare/10-259	0.122	0.991
013OpsBrbe/5-258	0.382	0.087
021NeuDare/1-252	0.115	1.207
022NeuTagu/4-255	0.8	-0.825
023NeuGaga/4-255	0.59	-0.371
024NeuMumu/1-198	0.71	-0.577
025NeuRano/7-244	0.823	-0.864

029NeuCafa/1-252	0.742	-0.692
030NeuEqca/1-252	0.794	-0.796
027NeuPatr/1-252	0.846	-0.871
028NeuHosa/1-252	0.828	-0.86
026NeuBota/1-250	0.694	-0.542
031NeuOran/1-252	0.737	-0.708
032NeuModo/1-252	0.726	-0.66
173RHOMumu/1-247	0.309	0.362
170RHOOrcu/1-247	0.52	-0.158
* 156RHOLeja/1-247	0.045	1.893
158RHOSasa/1-247	0.576	-0.312
148RHOGaga/1-247	0.21	0.687
135SWSOrla/1-247	0.447	0.046
208SWSTagu/46-291	0.052	1.752
204SWSNefo/1-247	0.223	0.637
217BluTaru/44-289	0.138	1.057
211SWSDare/45-290	0.631	-0.441
128SWSPore/31-276	0.14	1.109
126SWSOrla/31-275	0.226	0.614
059LWSGeau/18-264	0.796	-0.875
* 043uppTeni/1-245	0.011	3.053
054VAoDare/1-250	0.135	0.884
041ParXetr/1-250	0.255	0.502
233TMTDare/1-227	0.287	0.442
228EncDare/1-247	0.253	0.561
Plos1_1/19-255	0.547	-0.269
Plos1_3/1-234	0.294	0.348
Plos1_2/1-247	0.072	1.529
NV_CN158Suga08_/1-24	0.081	1.629
NV_CN152Suga08_/1-24	0.409	0.053
OG50DappuT/1-253	0.054	1.717
OG45ApimeT/17-269	0.083	1.392
NV_CN144Suga08_/1-23	0.427	0.01
NV_CN145Suga08_/1-23	0.542	-0.198
NV_CN137Suga08_/1-24	0.975	-1.548
NV_CN143Suga08_/1-24	0.323	0.264
NV_CN135_Suga08/1-24	0.239	0.517
ClR_CN108Suga08/1-19	0.409	0.085
CL_CN168_A9cr40/1-23	0.739	-0.71
ClR_CN116Suga08/1-23	0.717	-0.687
ClR_CN120Suga08/1-23	0.547	-0.24
HM_CN131_221128/1-20	0.303	0.396
HM_CN170_UPI000/1-23	0.079	1.518

ClR_CN101Suga08/1-24	0.319	0.285
CR_CN100_Koyana/1-24	0.174	0.721
TRA_530235_jgi|/1-22	0.588	-0.346
Tra_435785jgi|T/1-22	0.583	-0.341
MTR1C_XENL/7-188	0.257	0.532
UPI0001560/4-204	0.196	0.744
MLT_B2Y4M8_/1-198	0.131	1.177
MLT_O88495_/1-202	0.393	0.15
Tra_434217jgi|T/1-22	0.45	0.018
Tra_435667jgi|T/1-22	0.061	1.839
Tra_435668jgi|T/1-21	0.389	0.15
* Tra_435650jgi|T/1-22	0.02	2.613
* Tra_435652jgi|T/1-21	0.004	3.505
Tra_435654jgi|T/1-21	0.289	0.337
* Tra_435653jgi|T/1-21	0.036	2.077
Tra_435656jgi|T/1-21	0.063	1.634
* Tra_435655jgi|T/1-22	0.002	5.761
Tra_435633jgi|T/1-20	0.461	-0.134
Tra_435634jgi|T/1-16	0.108	1.109
* Tra_435635jgi|T/1-22	0.002	5.773
NEM_426957_jgi|/1-22	0.699	-0.586
NEM_445570_jgi|/1-22	0.769	-0.779
NEM_444070_jgi|/1-22	0.352	0.209
Mnemiopsis_opsin3_21	0.911	-1.177
Pleurobrachia_opsin2	0.832	-0.882
Mnemiopsis_opsin2_12	0.678	-0.555
Mnemiopsis_leidy1/1-	0.192	0.821
Pleurobrachia_opsin1	0.805	-0.845
acropsin2/1-248_no_c	0.11	1.33
acropsin1/1-240_no_c	0.162	0.964
acropsin3/1-252_no_c	0.886	-1.107

This table illustrates that few sequences in the data set are compositionally heterogeneous (indicated with an asterisk). An analysis has been performed Fig. S4 where these sequences were excluded to investigate whether the presence of heterogeneous sequences in the data affected our results. See the Phylobayes manual (Lartillot, Lepage, Blanquart 2009) for details about the posterior predictive test here performed, and the exclusion of compositionally heterogeneous sequences to investigate the effect of base composition bias on our results.

Supplementary Figures Captions.

Fig. Supplementary 1. Posterior predictive analysis of saturation. Posterior predictive analyses performed under (A) GTR + G, and (B) WAG + G. In blue: Predicted homoplasy. In red: Observed homoplasy. This figure clearly illustrates that under WAG + G the homoplasy in the data is systematically underestimated. This indicates the WAG + G model does not adequately fits Opsin data.

Fig. Supplementary 2. Results of the analyses of SEAm1 and SEAm2 under WAG + G. (A) Melatonin receptors as the only outgroups; (B) Melatonin receptors and Placopsins as outgroups.

Fig. Supplementary 3. Results of the analysis of FEAm1 performed under WAG + G.

Fig. Supplementary 4. Principal component analysis of FEAm1.

Fig. Supplementary 5. Results of the analysis of FEAm1 performed under GTR + G, after the compositionally heterogeneous sequences were excluded.

Fig. Supplementary 6. Results of the analysis of FEAm1 performed under GTR + G covarion model.

References

Lartillot, N, T Lepage, S Blanquart. 2009. PhyloBayes 3: a Bayesian software package for phylogenetic reconstruction and molecular dating. Bioinformatics 25:2286-2288.

Fig. S1

(A)

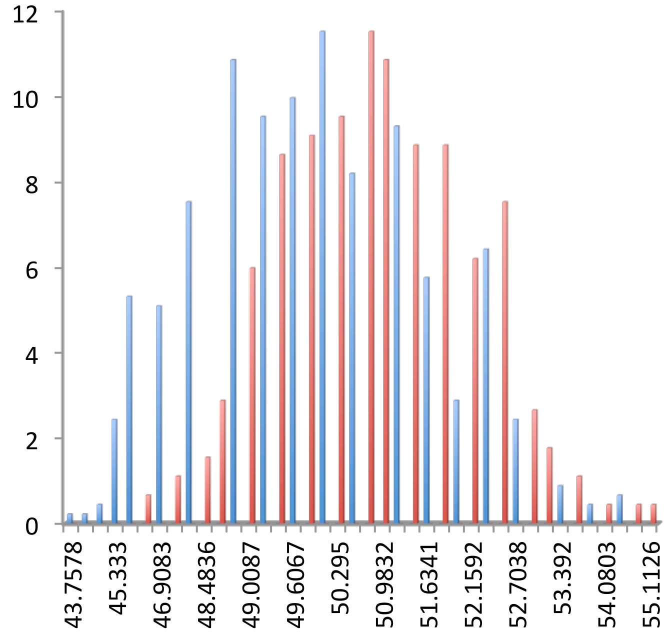
12
10
8
6
4
2
0
43.7578
45.333
46.9083
48.4836
49.0087
49.6067
50.295
50.9832
51.6341
52.1592
52.7038
53.392
54.0803
55.1126

(B)

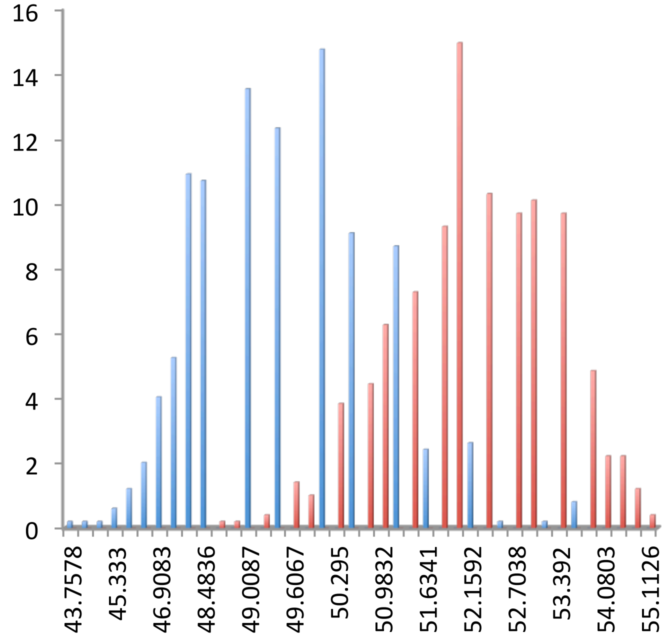
16
14
12
10
8
6
4
2
0
43.7578
45.333
46.9083
48.4836
49.0087
49.6067
50.295
50.9832
51.6341
52.1592
52.7038
53.392
54.0803
55.1126

Fig. S2

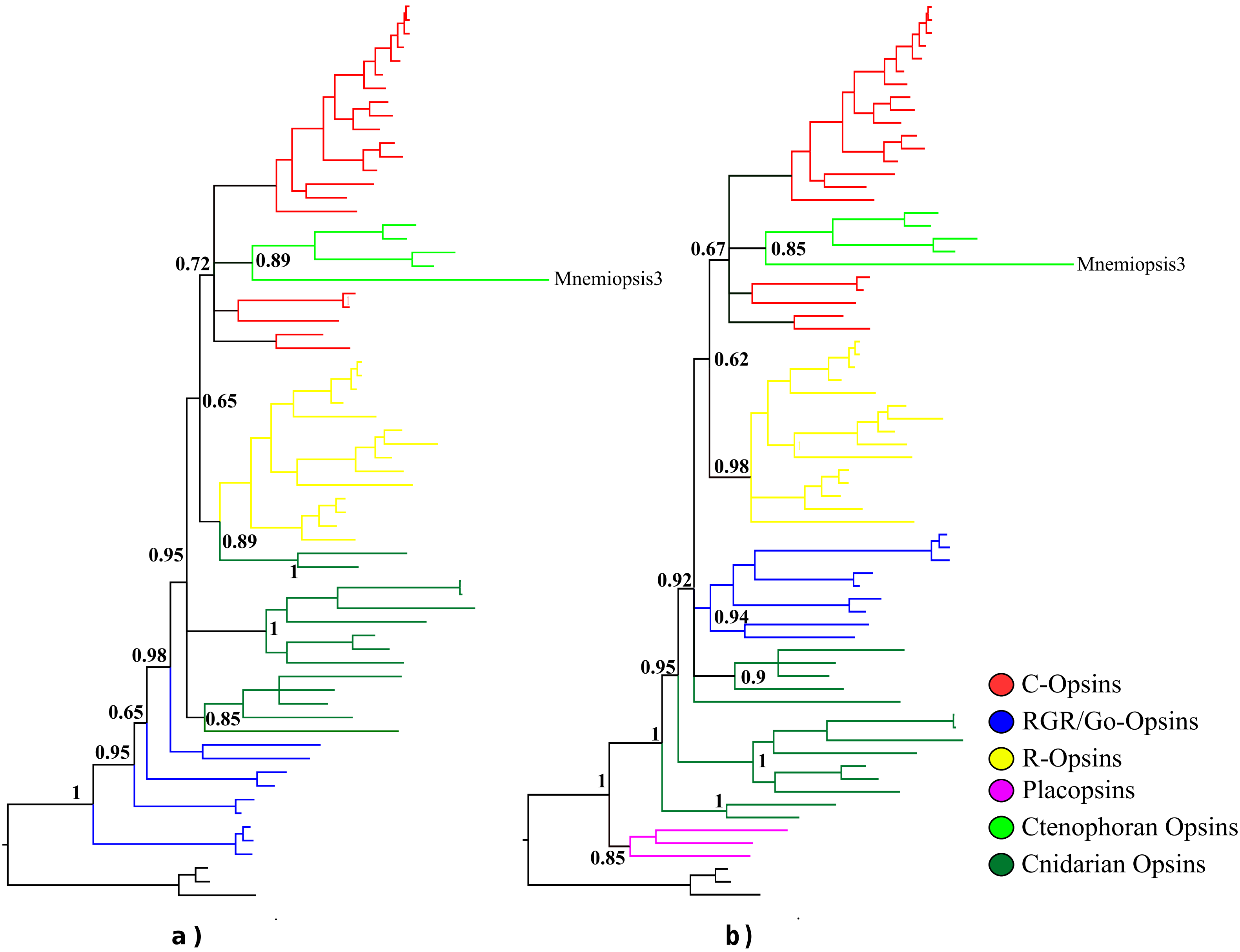
0.72
0.89
Mnemiopsis3
0.65
0.89
1
0.95
1
0.98
0.85
0.65
0.95
1
a)
0.67
0.85
Mnemiopsis3
0.62
0.98
0.92
0.94
0.95
0.9
1
1
1
1
0.85
b)
C-Opsins
RGR/Go-Opsins
R-Opsins
Placopsins
Ctenophoran Opsins
Cnidarian Opsins

Fig. S3

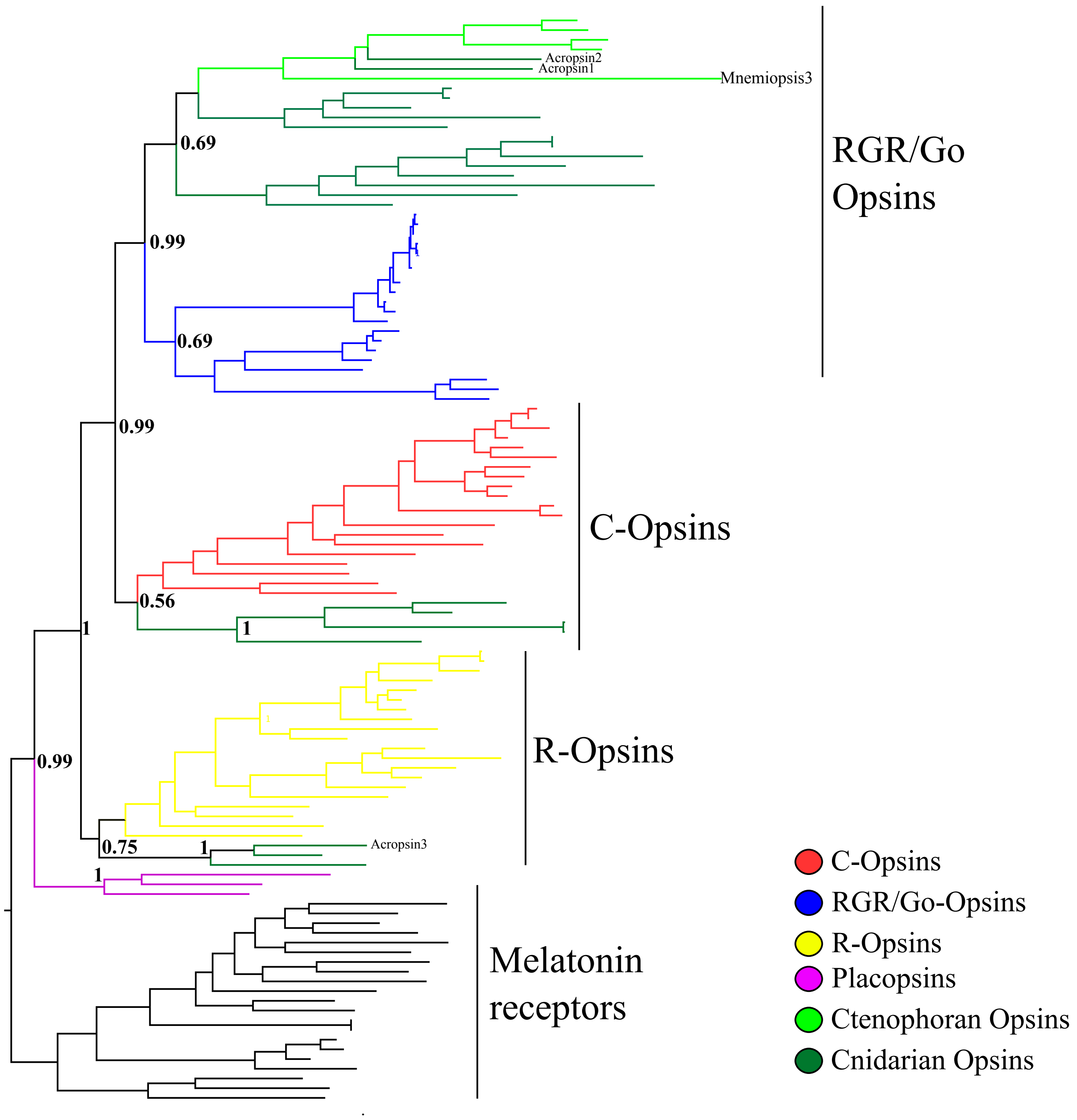

Acropsin2
Acropsin1
Mnemiopsis3
0.69
0.99
0.69
0.99
0.56
1
1
0.99
0.75
1
Acropsin3
1
RGR/Go Opsins
C-Opsins
R-Opsins
Melatonin receptors
C-Opsins
RGR/Go-Opsins
R-Opsins
Placopsins
Ctenophoran Opsins
Cnidarian Opsins

Fig S4

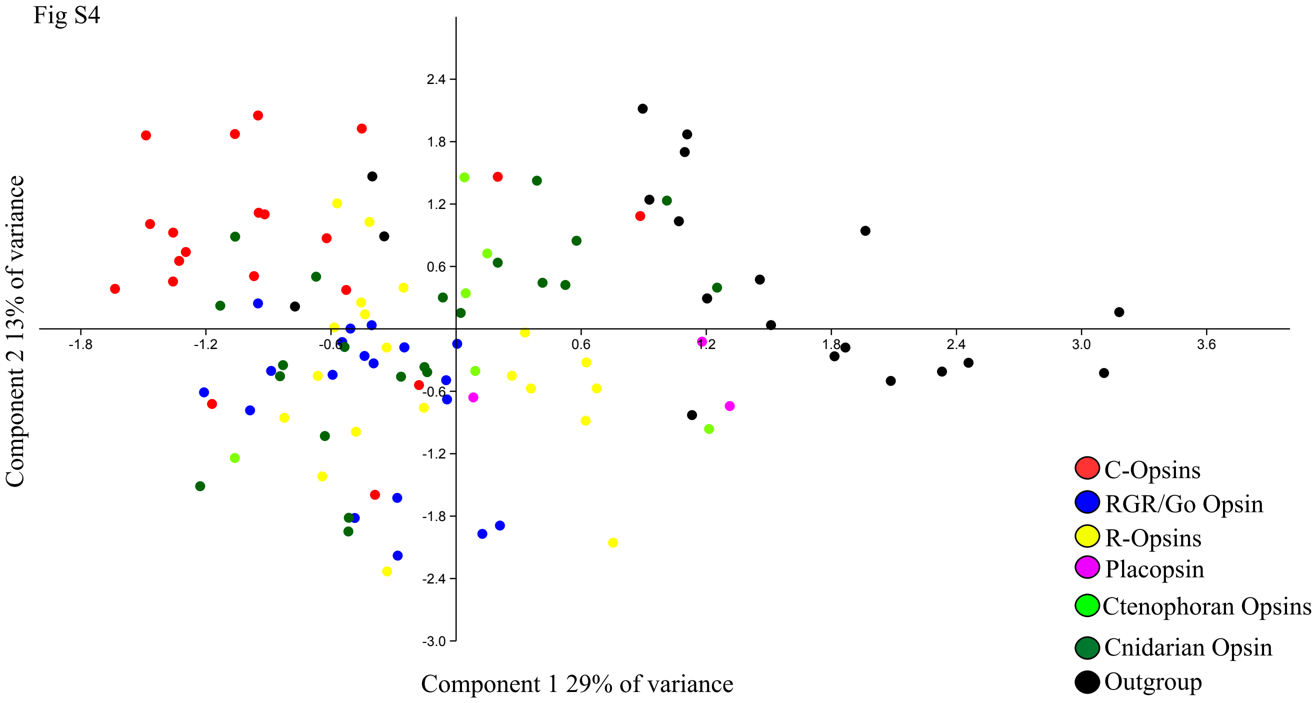

2.4
1.8
1.2
0.6
-1.8
-1.2
-0.6
0.6
1.2
1.8
2.4
3.0
3.6
-0.6
-1.2
-1.8
-2.4
-3.0
Component 2 13% of variance
Component 1 29% of variance
C-Opsins
RGR/Go Opsin
R-Opsins
Placopsin
Ctenophoran Opsins
Cnidarian Opsin
Outgroup

Fig. S5

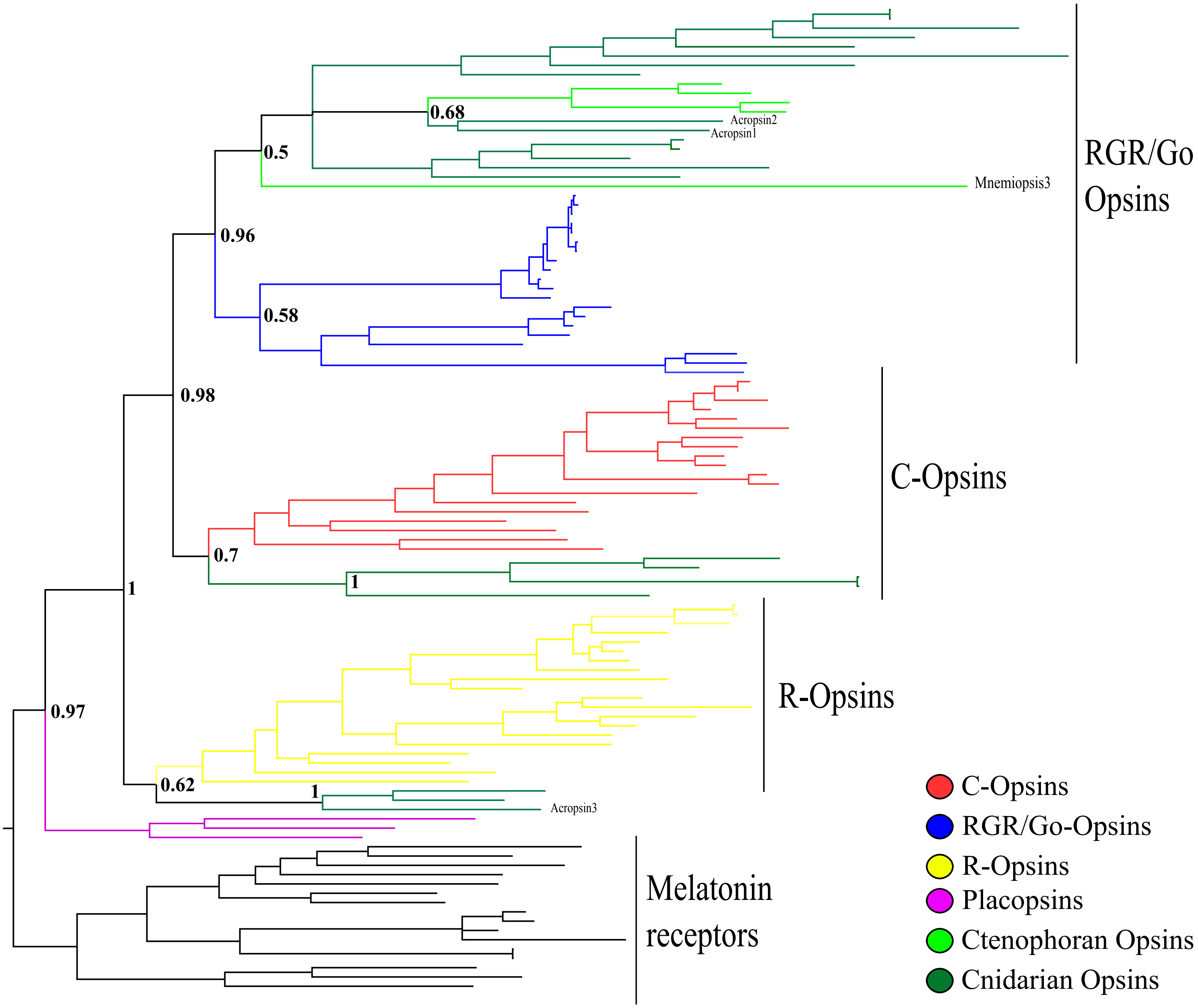

0.68
Acropsin2
Acropsin1
0.5
Mnemiopsis3
RGR/Go Opsins
0.96
0.58
0.98
C-Opsins
0.7
1
1
R-Opsins
0.97
0.62
1
Acropsin3
Melatonin receptors
C-Opsins
RGR/Go-Opsins
R-Opsins
Placopsins
Ctenophoran Opsins
Cnidarian Opsins

Fig S6

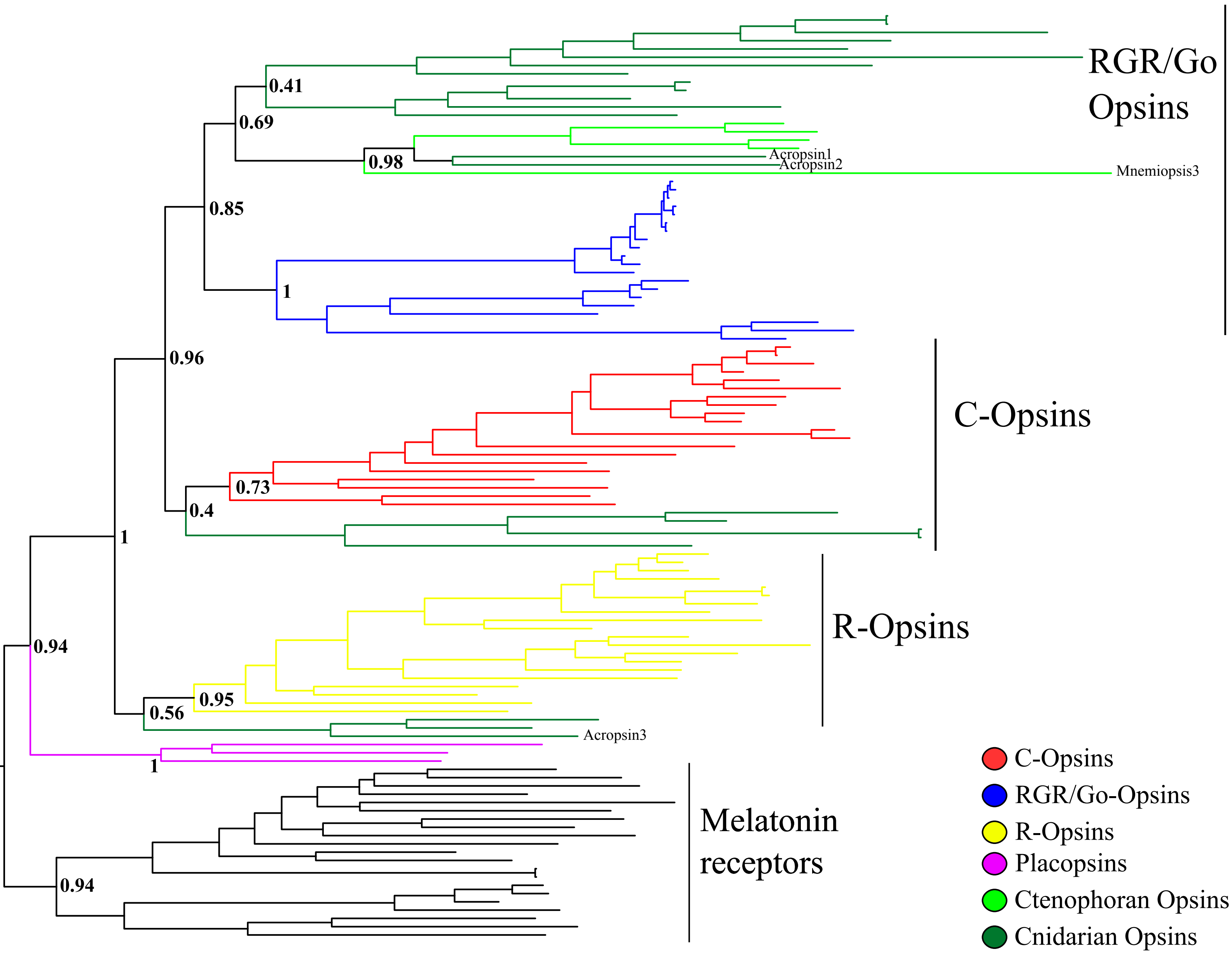
RGR/Go Opsins
C-Opsins
R-Opsins
Melatonin receptors
Acropsin1
Acropsin2
Mnemiopsis3
Acropsin3
0.41
0.69
0.98
0.85
1
0.96
0.73
0.4
1
0.94
0.95
0.56
1
0.94
C-Opsins
RGR/Go-Opsins
R-Opsins
Placopsins
Ctenophoran Opsins
Cnidarian Opsins
